# Considerations from the organoid perspective of hepatocytes: culture organoid of bone or cartilage

**DOI:** 10.1186/s12967-024-05081-2

**Published:** 2024-05-16

**Authors:** Xiaofei Wang, Jihang Dai, Wenyong Fei, Hengjun Gao, Jingcheng Wang

**Affiliations:** 1 Dalian Medical University, Dalian, China; 2 Department of Orthopedics, Northern Jiangsu People’s Hospital Affiliated to Yangzhou University, Yangzhou, China; 3 Shanghai National Engineering Research Center of Biochip, Shanghai, China; 4 Shanghai Engineering Center for Molecular Medicine, Shanghai, China

**To the editor,**

Organoids are miniaturised organ models that are developed from stem cells or tumour tissues extracted from patients in a specific 3D in vitro microenvironment and highly mimic the characteristics of real organs [1, 2].Although organoids are not human organs in the true sense of the word, they are able to mimic the structure and function of organs in the body to the greatest extent possible, and can be cultured for long-term stability.Many organoids have been generated from mouse and human stem cells, including intestines, kidneys, livers, brains, and retinal organoids, especially in the direction of cancer research [3, 4].

Vlachogiannis G et al. [5] found that patient-derived organoid models had a sensitivity of 100%, specificity of 93%, positive predictive value of 88%, and negative predictive value of 100% for predicting patient response to targeted drugs or chemotherapy, which is important for drug screening therapy.Conventional 2D cell culture is unable to reflect real biological processes due to its lack of hierarchical structure, dimensionality, cellular diversity and cell–cell or cell-substrate interactions, which makes it difficult to mimic cellular functions present in tissues.Organoids offer advantages over 2D cell culture such as closer to physiological cellular composition, a more stable genome and high throughput screening.Organoid technology enables the reconstruction of human organs and diseases in a petri dish for translational applications. Organoid models are simpler to manipulate than animal models and can also be used for mechanistic studies such as disease onset and progression.For example, organoid models can provide extensive biomedical utility in further understanding the biology of liver cancer and developing personalised medical approaches to the disease [6].

Mouse hepatocyte organoids preserve liver function and mimic hepatocyte physiology in an in vivo mouse model.Using a 3D culture system to produce mouse hepatocyte organoids, a method that more effectively mimics the in vivo environment, can provide a more physiologically relevant environment for studying liver function and response.Single cell shapes were observed when hepatocytes could be isolated from mouse livers and embedded in Matrigel. After 1–2 days of experience, this shape changes to a cluster or hollow shape and eventually becomes larger.

When the organoids were embedded in Matrigel and cultured for 1 week, the shape changed to a larger hollow shape or transformed to a solid state, and then reproduction for culture (Fig. 1).

**Fig. 1:**
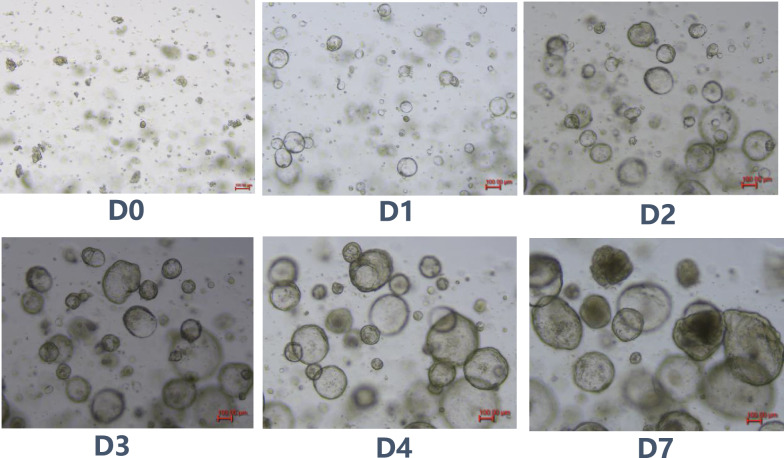
Microscopic images of organoids from 6th generation mouse hepatocytes. After isolation of primary hepatocytes from 8-week-old mice, which underwent stable passaging culture in Matrigel 3D cultures, the organoids of 6th generation mouse hepatocytes changed their morphology over time on day 0, day 1, day 2, day 3, day 4 and day 7

Organoid application scenarios are vast, and highly simulated disease models of organoids are expected to continue to make new progress in precision medicine, regenerative medicine and other fields.Different from the intestines, liver, kidneys and other organoids, although a variety of soft tissue organoids have been successfully constructed, the construction of bone organoids is still in its infancy.Bone organoids have three-dimensional spatial characteristics, which require precise regulation of osteoblast bone matrix secretion and mineralisation, as well as their correct arrangement at the spatial level, thus presenting many difficulties, but this research direction has high clinical value.

For example, cartilage organoids can be self-assembled from human pluripotent stem cells into cartilage organoids for the treatment of bone and joint pathologies such as osteoarthritis or cartilage defects, in order to solve the many degenerative joint pathologies brought about by aging, and to provide an important basis for drug screening and selection of therapeutic options for orthopaedic diseases. Along with the development of organoid technology, organoid chips, as an important development direction of organ chips, will also promote the development of bone organoids.

However, the research and application of organoids is still at an early stage, and the stability and efficiency of the organoid technology itself has yet to be improved and optimised.It should be noted that it is relatively easy to build a single unit of bone organoid, but achieving multifunctionality in a complete bone organoid is a difficulty that needs to be solved at present. Moreover, the current organoid construction of bone needs to solve many difficulties of matrix scaffold materials, cell differentiation regulation methods, and vascularisation techniques.

It is believed that in future research, bone organoids will be transferred from basic research to clinical application, which will accelerate the development of bone tissue engineering technology on one hand, and on the other hand, large-size bone organoids can be directly used for bone defect repair and bone regeneration. This can provide functional and personalised clinical solutions for patients.

## Data Availability

All data for this study are publicly available.
